# In the June 2011 issue

**DOI:** 10.1590/S1980-57642011DN05020001

**Published:** 2011

**Authors:** Ricardo Nitrini

**Affiliations:** Editor-in-Chief

In this issue we are publishing one review, 10 original articles, one case report and the section News & Perspectives.

**Spindola and Brucki** reviewed the currently available literature on prospective memory in Alzheimer’s disease (AD) and mild cognitive impairment (MCI). The authors found that both AD and MCI patients show important impairment of prospective memory, but the accuracy of prospective memory tests in the early diagnosis of these conditions is still undefined.

**Balthazar et al.** investigated the neuroanatomical correlations of naming and semantic generalization in a group of individuals including normal elders and patients with amnestic MCI and AD, using the Boston naming test and voxel-based morphometry to measure whole brain gray matter density. The areas more related to naming and semantic generalization were identified and their possible role in lexical-semantic networks is discussed.

**Venegas and Mansur** evaluated the number of items generated in verbal fluency tests by the elderly. Most of the items were produced in the first 15 seconds of this one minute-test, for both phonemic and semantic tests. The authors concluded that an abbreviated version of the verbal fluency test may be an interesting proposal for the clinical evaluation.

**Charchat-Fichman et al.** studied verbal fluency, semantic and phonemic, in a group of 119 children (7 to 10 years-old). The authors found that the effect of age was significant for both tasks, and a significant difference was found between the 7 and the 9 year-old subjects, or between the 7 and the 10 year-old subjects, whereas the 8-year-old group was not different from the other age groups of the study.

**Batistoni et al.** investigated the effects of the participation in social, educational and leisure activities on the prevalence of depression in the elderly. Among elderly of an Open University for the Third Age, participation time over one semester was associated with less depressive symptoms when compared with those participating for less than one semester.

**Fabricio and Yassuda** compared the memory strategies used by young and old adults using several different memory tests. Although young adults outperform seniors, both groups reported the use of similar strategies.

**Diaz et al.** compared the cognitive performance of two groups of long term institutionalized elderly patients: one with schizophrenia and the other with Hansen’s disease. Patients with schizophrenia showed a worse performance in multiple cognitive domains.

**Matioli et al.** evaluated the worries about memory loss and knowledge about AD using a simple questionnaire in a community-dwelling elderly. The knowledge about AD and on the meaning of memory decline was poor, reinforcing that the need of strategies to provide more information on these topics to the elderly.

**Cachioni et al.** evaluated caregivers of AD patients who were participating of support groups. The authors found that caregivers were mostly women, and that the level of emotional burden was not as high as it was expected, possibly due to the participation in support groups.

**Ribeiro et al.** investigated the level of attention in deaf persons who work with computers. The comparison was made with normal controls that use computers, normal controls that do not use computers, and deaf persons that do not use computers. The authors concluded that persons deaf from birth who work with computers presented higher levels of attention, sustained attention and resistance to interference when compared to the other three groups.

**Teixeira et al.** used the Cambridge Neuropsychological Test Automated Battery (CANTAB) to evaluate the spatial memory span of Brazilian children and adolescents. Their findings were similar to those reported for Australian children and adolescents, a finding that points to the adequacy of this task for assessment of working memory in Brazilian children.

**Brito-Marques et al.** investigated the case of a patient with histopathologic-proven corticobasal degeneration that presented with progressive non-fluent aphasia. The authors concluded that this type of clinical progression to corticobasal degeneration reflects a anatomical pattern of cortical involvement.

Finally, in the section News & Perspectives, **Brucki** presented brief comments on relevant and recently published papers.

**Ricardo Nitrini** Editor-in-Chief

